# Ectogenic tension promotes fibrogenesis of mesenchymal stem cells through microRNA-21

**DOI:** 10.1038/cddiscovery.2016.99

**Published:** 2017-04-10

**Authors:** Wei Cao, Bo Ou, Yufang Shi

**Affiliations:** 1 Key Laboratory of Stem Cell Biology, Institute of Health Sciences, Chinese Academy of Sciences, 320 Yueyang Road, Shanghai 200031, China; 2 Hainan West Central Regional Hospital, 2 East Fubo Road, Danzhou 571700, China; 3 Jiangsu Key Laboratory of Stem Cells and Medicinal Biomaterials and The First Affiliated Hospital of Soochow University, Institutes for Translational Medicine, Soochow University, 199 Renai Road, Suzhou 215123, China

Dear Editor,

Adult stem/progenitor cells are imperative for tissue repair, regeneration and cellular homeostasis. When the equation of new cell generation and steady-state cell loss is perturbed by tissue injury, stem cells are mobilized to participate in reconstruction of the injured tissue through their replication and differentiation. As a type of progenitor cells, mesenchymal stem cells (MSCs), because of their broad tissue distribution, multipotent differentiation capacity and extraordinary immunomodulatory property, are believed to have critical roles in tissue regeneration.^1^ Attracted by factors released from dying/dead cells and inflammatory cells, MSCs migrate specifically to damaged tissue sites and actively modulate the local inflammatory microenvironment. In addition, upon encountering inflammatory factors, MSCs release large amounts of various chemokines and growth factors, which in turn attract inflammatory cells and activate local intrinsic tissue stem cells.^2^ These cells will clean up the damaged tissue sites and differentiate into cells needed for repairing damaged tissues. It is important to point out that it is not known whether MSCs are recruited from bone marrow or from tissues of close vicinity; nevertheless, they become fully exposed to the cells, metabolites, soluble factors, extracellular matrix (ECM) and mechanical forces in damaged tissue. The interplay between MSCs and the surrounding tissue microenvironment inevitably determine the reparation outcome. Therefore, defining the complicated interplays between MSCs and the tissue microenvironment will help facilitate manipulations of MSC fate for better MSC-based tissue repair and/or regeneration.

Tissue reparation can be accompanied by sustained injury signals such as chronic inflammation and dynamic changes in tissue mechanical stiffness. Indeed, it is becoming increasingly accepted that mechanical cues inherent to the stiff fibrotic ECM are as important as chemical composition in regulating cell behaviors.^3^ Interestingly, various cell types have been found to possess ‘mechanical memory’, a cellular property believed to be permanently imprinted for memorizing the substrate mechanical conditions.^4^ Although naïve MSCs have been demonstrated to specify lineage and commit to phenotypes with extreme sensitivity to tissue elasticity, the mechanical memory has also been demonstrated to influence lineage choice of MSCs on a shorter timescale.^5^ However, whether substrate-imprinted mechanical memory affects the fibrogenic behavior of cultured MSCs and whether mechanical memory can be manipulated to enhance the success of MSC-based therapy remains to be determined.

In the most recent issue of *Nature Materials* Dr. Boris Hinz’s group presented their novel insight into the molecular mechanisms that regulate long-term mechanical memory that affects the fibrogenic programs of cultured MSCs.^6^ In their first sets of experiments, Chen *et al.* initiated rat MSC cultures directly on soft and stiff silicone rubber substrates and characterized these cells. Consistent with previous reports, the authors found that culturing on stiff substrates stimulated MSC fibrogenesis and osteogenesis, whereas soft substrates reduced the fibrogenesis potential of MSCs, accompanied by high clonogenicity and multipotency. In fact, MSCs has been demonstrated to possess mechanosensing and transducing machineries, which intensely affects MSC behaviors.^7^ However, whether MSCs acquire long-lasting mechanical memory in response to different substrate culture priming is unknown. In this study, the authors cultured MSCs on soft and stiff silicone substrates for three passages before switching to the respective substrate stiffness for additional two passages. It was found that the mechanical memory was acquired by MSC priming on pathophysiologically stiff/soft substrate, and retained for at least two passages, regardless of the subsequent mechanical stimuli. Furthermore, the authors established a rodent model of skin wound healing to test whether the acquired mechanical memory in MSCs persists after topical transplantation. It was demonstrated that MSCs priming on soft substrates improved wound healing quality characterized by reduced granulation tension, reduced myofibroblast content, decreased collagen density and increased angiogenesis compared with that of control and stiff-primed groups. Conversely, stiff-primed MSCs memorized their culture activation force and demonstrated enhanced scarring *in vivo*. Although cell-culture-established mechanical memory offered a robust activity against proinflammatory and profibrotic conditions in injured tissue, it is not clear how mechanically primed MSCs affected wound quality. As MSCs produce various factors, such as angiopoietin-1, vascular endothelial growth factor, epidermal growth factor, fibroblast growth factor and transforming growth factor-*β* (TGF-*β*), which directly affect various cells including endothelial cells and fibrobasts.^8^ It is conceivable that primed MSCs may exert trophic effects on various resident cell populations. Given that MSCs possess potent immunomodulatory activity, it is interesting to further investigate whether mechanical priming has impacts on the immunomulatory properties of MSCs.

The authors next investigated the molecular mechanism underpinning the long-term mechanical memory of the fibrogenic program in MSCs. By screening a series of fibrosis-related genes, the authors identified microRNA-21 (miR-21) as a central regulator of mechanical memory of MSCs (Figure 1). The levels of miR-21 was found to increase progressively over five passages of MSCs on stiff substrates, but remained constitutively low on soft substrates. The next question is how substrate mechanics affect miR-21 expression. Based on the presence of one conserved CArG box in the miR-21 promoter, which potentially serves to bind serum response factor in conjunction with myocardin-related transcription factor-A (MRTF-A), the authors investigated the alteration in MRTF-A activities. Interestingly, MRTF-A was found to respond acutely to stiff substrates by increased translocation into the nucleus, where MRTF-A binds to the CArG box to control transcription of miR-21. The subsequent ‘gain-of-function and loss-of-function’ experiments verified the role of miR-21 in the memory acquisition. Transfecting soft-primed MSCs with miR-21 mimics resulted in increased *α*-smooth muscle actin (*α*-SMA) mRNA levels, whereas knockdown of miR-21 in stiff-primed MSCs reduced *α*-SMA mRNA levels. In addition, some markers of the fibrogenic cell program and the predicted direct targets of miR-21 were shown corresponding alterations in MSCs with the disruption of miR-21 levels. MiR-21 has been reported to regulate the MSC osteogenic lineage fate.^9^ Consistently, overexpression of miR-21 in soft-primed MSCs facilitated osteogenic differentiation and downregulation of miR-21 in stiff-primed MSCs enhanced MSC adipogenesis, suggesting that the onset of a fibrotic program alters the MSC lineage choice.

Surprisingly, the authors found that the levels of miR-21 maintained for at least two passages of MSCs after substrate stiffness switching from stiff to soft, and even knockdown of MRTF-A in stiff-primed MSCs had no effect on miR-21 levels up to 4 days thereafter. In contrast, MRTF-A activity fluctuated acutely responding to changes of mechanical stiffness. The authors then determined the mechanisms controlling the maintenance miR-21 levels. As the miR-21 replenishment to soft condition-primed MSCs exhibited continuous fibrogenic activity, it is possible that there is a mechanism to keep the miR-21 stability. To verify whether established miR-21 levels indeed preserved mechanical memory, the authors disrupted miR-21 levels in mechanical-primed MSCs before substrate switching. It was found that downregulation of miR-21 expression in stiff-primed MSCs completely abrogated the stiff-priming effects and rendered the cells susceptible to a phenotype of soft substrate primed, as indicated by reduced *α*-SMA levels and decreased profibrotic protein expression, and *vice versa*. Furthermore, the role of miR-21 as the mechanical memory keeper was confirmed in the rat wound healing model. Application of stiff-primed MSCs with erased mechanical memory (downregulation of miR-21 with antagomirs) was found to attenuate scarring formation, an effect comparable to that of soft-primed MSCs. Therefore, priming on physiologically soft substrates and/or erasing mechanical memory will protect MSCs from becoming profibrotic *in vivo* and promote MSC-mediated tissue repair.

Taken together, the findings of Chen *et al.* provide novel insights into the fibrotic mechanical memory of MSCs and reveal potential novel strategies that may improve therapeutic outcome for MSC-based tissue engineering. In the context of tissue injury, MSCs delivered to damaged tissue sites have extensive interaction with surrounding microenvironment including infiltrated immune cells and inflammatory mediators. It is becoming clear that MSC-mediated immunomodulation is prominent in orchestrating local inflammatory microenvironment and facilitating the tissue repair activity of intrinsic tissue stem cells. Prompted by the finding that MSCs preserve mechanical memory, we come to question whether MSCs have similar ‘immunomemory’ properties. In fact, the concept of a plasticity of MSC-mediated immunomodulation depending on the inflammatory status is becoming highly relevant.^10^ Pretreatment with proinflammatory cytokines such as IFN-*γ* and TNF-*α* has been demonstrated to promote MSC-mediated therapy in some inflammatory disorders.^11,12^ Similar to mechanical memory, cytokine-primed MSCs seem to acquire ‘immunomemory’ according to the microenvironment conditions *in vivo*. We believe that studies on MSC memory will shed new lights on MSC-based cell therapy not only in tissue repair applications but also in immune-related therapies.

## Figures and Tables

**Figure 1:**
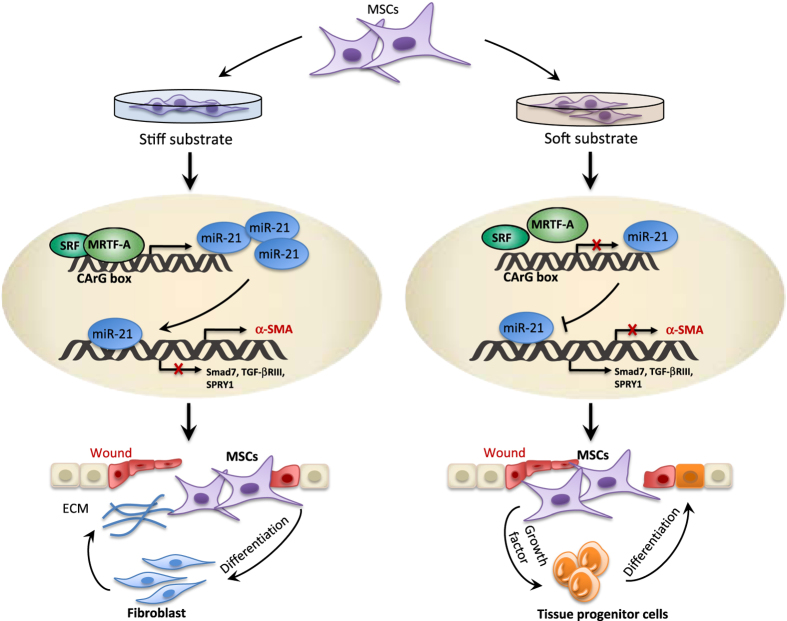
The role of miR-21 in the long-lasting mechanical memory of MSCs in response to ectogenic tension. When MSCs cultured on stiff substrates, MRTF-A is induced to translocate to the nucleus where it binds to the CArG box to promote transcription of miR-21. High levels of miR-21 is maintained for a long term through unknown mechanisms. The persistent presence of miR-21 promotes the transcription of *α*-SMA and inhibits the transcription of antifibrogenic genes including Smad7, TGF-*β* receptor type III (TGF-bR III) and sprouty homolog 1 (SPRY1). Stiff-primed MSC memorizes their culture activation force and acquire fibrogenic capability *in vivo*. Conversely, when MSCs were cultured on soft substrates, the miR-21 level is kept low and thus reduce the profibrotic activity *in vivo* and allows better MSC-mediated tissue repair.
